# New Approaches to the Integration of Navigation Systems for Autonomous Unmanned Vehicles (UAV)

**DOI:** 10.3390/s18093010

**Published:** 2018-09-08

**Authors:** Ivan Konovalenko, Elena Kuznetsova, Alexander Miller, Boris Miller, Alexey Popov, Denis Shepelev, Karen Stepanyan

**Affiliations:** Institute for Information Transmission Problems RAS, Bolshoy Karetny per. 19, build.1, Moscow 127051, Russia; konovalenko@iitp.ru (I.K.); kuznetsova@iitp.ru (E.K.); amiller@iitp.ru (A.M.); ap@iitp.ru (A.P.); shepelev@iitp.ru (D.S.); KVStepanyan@iitp.ru (K.S.)

**Keywords:** UAV, videonavigation, projective geometry, feature points, Kalman filter

## Abstract

The article presents an overview of the theoretical and experimental work related to unmanned aerial vehicles (UAVs) motion parameters estimation based on the integration of video measurements obtained by the on-board optoelectronic camera and data from the UAV’s own inertial navigation system (INS). The use of various approaches described in the literature which show good characteristics in computer simulations or in fairly simple conditions close to laboratory ones demonstrates the sufficient complexity of the problems associated with adaption of camera parameters to the changing conditions of a real flight. In our experiments, we used computer simulation methods applying them to the real images and processing methods of videos obtained during real flights. For example, it was noted that the use of images that are very different in scale and in the aspect angle from the observed images in flight makes it very difficult to use the methodology of singular points. At the same time, the matching of the observed and reference images using rectilinear segments, such as images of road sections and the walls of the buildings look quite promising. In addition, in our experiments we used the projective transformation matrix computation from frame to frame, which together with the filtering estimates for the coordinate and angular velocities provides additional possibilities for estimating the UAV position. Data on the UAV position determining based on the methods of video navigation obtained during real flights are presented. New approaches to video navigation obtained using the methods of conjugation rectilinear segments, characteristic curvilinear elements and segmentation of textured and colored regions are demonstrated. Also the application of the method of calculating projective transformations from frame-to-frame is shown which gives estimates of the displacements and rotations of the apparatus and thereby serves to the UAV position estimation by filtering. Thus, the aim of the work was to analyze various approaches to UAV navigation using video data as an additional source of information about the position and velocity of the vehicle.

## 1. Introduction

The integration of observation channels in control systems of objects subjected to perturbations and measurement errors of the motion is based on on the observations control theory started in the early 1960s. The first works on this topic were based on the simple Kalman filter property, namely: the possibility of determining the root-mean-square estimation error in advance, without observations, by solving the Riccati equation for the error covariance matrix [1]. The development of this methodology allowed solving problems with a combination of discrete and continuous observations for stochastic systems of discrete-continuous type. At the same time, methods were developed for solving problems with constraints imposed on the composition of observations, temporal and energy constraints both on separate channels and on aggregate. For a wide class of problems with convex structure, necessary and sufficient conditions for optimality were obtained, both in the form of dynamic programming equations and the generalized maximum principle, which opens the possibility of a numerical solution [2,3]. The tasks of integrating surveillance and control systems for UAVs open a new wide field of application of the observation control methods, especially when performing autonomous flight tasks. One of the most important problems is the detection of the erroneous operation of individual observation subsystems, in which the solution of navigational tasks should be redistributed or transferred to backup subsystems or other systems operating on other physical principles [4].

A typical example: navigation through satellite channels such as global positioning system (GPS), which is quite reliable in simple flight conditions, but in a complex terrain (mountains, gorges), it is necessary to use methods to determine your position with the help of other systems based, for example, on landmarks observed either with optoelectronic cameras, or radar [5,6,7,8]. Here the serious problem of converting the signals of these systems into data suitable for navigation arises. The human-operator copes with this task on the basis of training. That is the serious problem in computer vision area and it is one of the mainstream in the UAV autonomous flight [9]. Meanwhile, the prospects for creating artificial intelligence systems of this level for UAV applications are still far from reality.

At the same time, the implementation of simple flight tasks, such as either access to the aerial survey area or tracking the reference trajectory [10,11,12] the organization of data transfer in conditions of limited time and energy storage [13,14] and even landing [15], are quite accessible for performing UAVs in the autonomous mode with reliable navigation aids.

Unmanned aerial, land-based and underwater-based vehicles that perform the autonomous missions use, as a rule, an on-board navigation system supplemented by sensors of various physical nature. At the same time, unlike remote control systems in which these sensors present information in the form as operator-friendly as possible, the measurement results should be converted into the input signals of the control system, which requires other approaches. This is especially evident in the example of an optical or optoelectronic surveillance system, whose purpose in remote control mode is to provide the operator with the best possible image of the surrounding terrain. At the same time, in an autonomous flight, the observing system should be able to search for the characteristic objects in the observed landscape and give the control system their coordinates and estimate the distances between them. Of course, the issue of providing an exellent image and determining the metric properties of the observed images are connected, and in no case cancel one another. However, what a human operator does automatically basing on a sufficiently high-quality image of the terrain, the readings of other sensors and undoubtedly on previous experience, the control system algorithm must do by using data from video and other systems, with the same accuracy as the human operator.

In this paper, various approaches to the integration of surveillance channels for UAVs are presented. Some of which were tested on real data obtained during flights with registration of the underlying earth’s surface by on-board video-camera. In our previous works [16,17] we examined the approach to video navigation using the methodology of detecting special “points”, the coordinates of which are predetermined on a reference image of the terrain formed on the basis of satellite or aerial imagery [12]. However, in the practical use of this method, difficulties were encountered in identifying these specific points. These difficulties are associated with a significant difference in the quality of the on-board and reference images with the essential difference in scales. Therefore, when using real images, the best results were obtained by matching images not by points, but by rectilinear segments, such as images of road sections, walls of buildings [18]. This algorithm is a new modification of the navigation for the “sparse” optical flow (OF). Moreover, in areas of non-conjugation of template and on-board images for tracing the trajectory of UAV, it is necessary to apply methods based on so-called “dense” OF with the determination of the angular and linear velocities of the camera.

In the next Section 2 we present a review of various approaches applied to a video navigation and tested during work performed by a research group joined in the IITP RAS. Then in Section 3 we consider various approach to filtering in estimation of the UAV position. The approach to navigation based on computing of projective matrices between successive frames registered by on-board camera presented with the joint filtering algorithm in Section 4. Section 5 is conclusion.

We should underline that the main contribution of the paper is a review of different approaches to the UAV video navigation along with results of some experiments related to possible new developments which look promising in implementation of long-term autonomous missions performed by multi-purpose UAV.

## 2. General Approach to the Data Fusion Model of UAV Navigation

### 2.1. UAV Motion Model

In modern conditions, it becomes extremely important to fulfill UAV missions without using external navigation systems, this is why in this paper we focus on the benefits which can be derived directly from video data and on how to convert them into the UAV control system entrance form. The autonomous UAV navigation tasks solution requires obtaining the coordinates estimates, camera orientation angles, as well as the coordinates velocities of the apparatus itself and the angular velocities of the camera orientation change. For navigation one can use simple UAV model taking into account kinematic relations for position, velocities and accelerations such as (1), which in discrete time have a form

(1) $$\begin{array}{c} X\left(t_{k+1}\right)=X\left(t_{k}\right)+V\left(t_{k}\right)\Delta t+\mathrm{ACC}\left(t_{k}\right)\frac{\Delta t^{2}}{2},\\ V\left(t_{k+1}\right)=V\left(t_{k}\right)+\mathrm{ACC}\left(t_{k}\right)\Delta t,\\ \mathrm{ACC}\left(t_{k}\right)=U\left(t_{k}\right)+W\left(t_{k}\right). \end{array}$$

Here $t_{k}$ is a sequence of discrete times, such that $t_{k+1}-t_{k}=\Delta t,$ $X\left(t_{k}\right)\in R^{3}$ is vector of current UAV coordinates in the Earth coordinate system, $V\left(t_{k}\right)\in R^{3}$ is velocities vector, $\mathrm{ACC}\left(t_{k}\right)\in R^{3}$ is accelerations vector of, $U(t_{k})$ is vector of programmed accelerations from the UAV control system, $W(t_{k})$ is a vector of perturbations including aerodynamic influences and control system errors.

For navigation needs we must get the current attitude and velocity estimates.

Thus, to solve the navigation problems, the motion model must be completed with a model of observations, which can contain, in an explicit or implicit form, the current coordinates and/or velocities and possibly accelerations. Typically, this information comes from the inertial navigation system (INS) and from the sensor system or the global satellite navigation system and serves as an additional means which increases the accuracy and detects failures in the navigation system. For autonomous UAV flights this additional system is highly required and if the trained pilot uses this information automatically, for UAV it is necessary to convert the video information into data suitable for use by the vehicle control system. Below we give a series of examples of video features used for navigation.

### 2.2. New Possibilities Related to the Usage of On-Board Opto-Electronic Cameras

The use of opto-electronic cameras aboard the UAV opens a multitude of ways to separately or jointly evaluate the coordinates and velocities that characterize the position of the UAV and the orientation of the surveillance system. Some examples of successful usage of on-board cameras for micro aerial vehicles (MAV) in GPS denied environment were reported in [19,20]. It is known the series of succesful usage of such small cameras in various applcations including indoors and outdoor MAV autonomous flights [21,22]. However, in this research we were focused on outdoor UAV applications with the usage of on-board camera as an additional source of navigation information. It should be noted that there are only two approaches to video navigation: the first one is navigation through ground objects with known coordinates and the second one which is determination of the absolute UAV velocities, by observing the evolution of the video image of the underlying surface. In both cases one needs to take into account the filtering of altitude and the speed of the device received from the INS. In reality, both approaches should be used, but it is necessary first to investigate their accuracy characteristics. These accuracy characteristics, of course, depend on a variety of factors, such as illumination, shooting conditions, seasonality and others. Therefore, it is not possible to determine in advance which algorithms and approaches will be most effective. This is what determines the purpose of this work—to review the existing methods and, if possible, to assess their effectiveness in video navigation issues.

We list only a few of them and give our comments related to our experience obtained with real and/or virtual flights.

- Usage of the terrain maps and comparing the images of observed specific objects with its position on a preloaded terrain map. This seemingly most obvious method requires the presence of huge collection of observable objects on the board for reliable operation of the recognition system. These images must be recorded under different observation conditions, including aspect, scale, lighting, and so on. Of course, for some characteristic objects these problems are completely surmountable, but on the whole this creates serious difficulties.
- To solve this problem, special techniques have been invented that can be attributed to the allocation of some characteristic small regions (singular points) that are distinguished by a special behavior of the illumination distribution that can be encoded by some set of features that are invariant to the scale and change of the aspect angles [23,24,25,26]. The application of this approach is described in the work [16,27], where it is demonstrated on model images using a 3D map of the local area. In these work we used a computer simulation of a UAV flight and simulated on board video camera imaging. The simulating program is written in MATLAB. Type of the feature points are: ASIFT realized in OpenCV (Python) [26]. Feature points in this model work as in a real flight because the images for the camera model and for the template images were transformed by projective mapping and created by observations from different satellites. However, the use of this method is limited by the need to ensure the closeness of registration conditions. Moreover, significant difference in the resolution level of the reference image and the recorded images in flight also leads to significant errors.
- In tasks of the UAV navigating with the use of a preloaded map of the ground, the matching of reference and observable images plays a fundamental role. In recent years, the methodology based on the images matching with the use of singular points has been further developed. For example ORB methodology versus SIFT and SURF, use a very economical set of binary features of singular points, which allows to significantly reduce the execution time of the registration operation and demonstrate very high resistance to images noise and rotations [28]. In a series of detailed surveys [29,30,31] various alignment methods are examined either on the Oxford dataset test set and on others, while the ORB performance is high in terms of time consuming and the rate of erroneous pixel matching. Meanwhile, from the viewpoint of solving the problems of video navigation, it is more important the accuracy of matching, and more importantly, for specific images such as aerial photographs. In this connection, the results obtained with photogrammetric surveys using ORB-SLAM2 [32,33,34] which show the high potentialities of the ORB methodology, are of great interest in applications related to video navigation.
- Less sensitive to the difference in shooting conditions are methods based on combining extended linear objects such as roads, house walls, rectilinear power lines and so on [18]. The application of analysis of linear objects gives rise to the usage of fast Hough transform [35,36].

**Figure 1:**
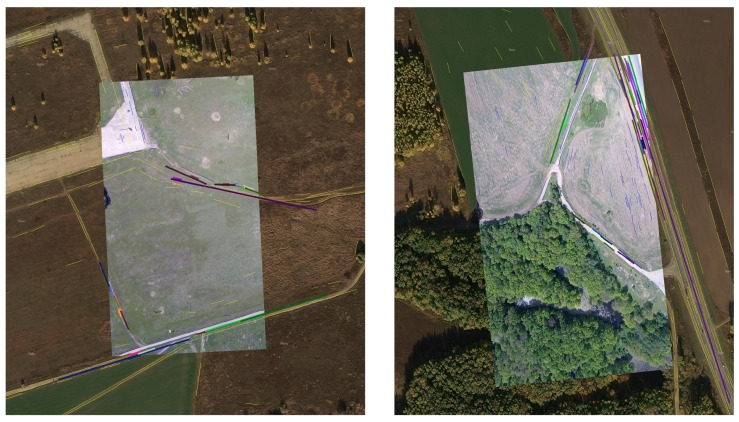
Left image shows the bad alignment of a template image and the on-board captured images. In this case the coordinates determined from current image are useless and not used for determining of the UAV coordinates. The right image shows the good alignment of template and the on-board captured images. One can observe the alignment of linear elements which are not connected, it shows the good information capability of this method.

Here we give some results of the image matching based on combining the linear objects (see Figure 1).

**Figure 2:**
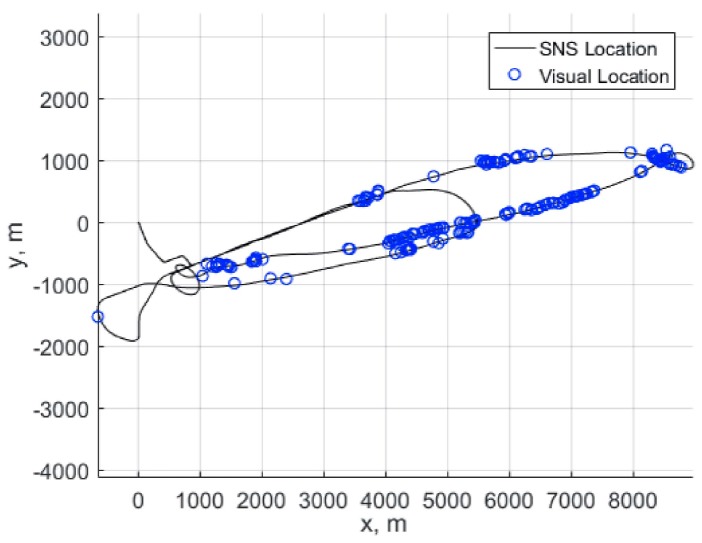
Solid line shows the position of the vehicle obtained from Satelite+ Navigation system (SNS). Blue circles show the points where the good alignment had been achieved.

The estimation of the trajectory, based on the alignment of linear elements on template and on-board captured image shows good quality of the vehicle position estimation (see Figure 2). Of course, in the areas where there is no alignments the position is estimated on the basis of INS data only.

**Figure 3:**
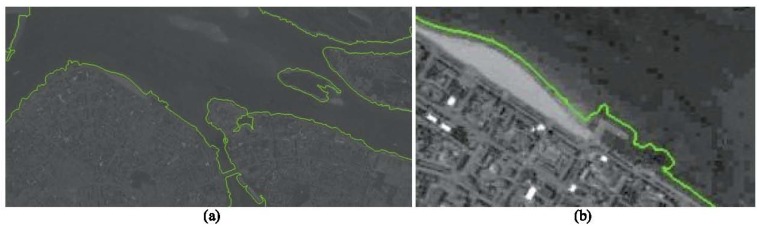
Texture segmentation results: (**a**) full image, (**b**) zoomed and contrasted image containing the coastal line.

- Similarly to linear objects, it is possible to use curvilinear shape preserving their forms for successful alignment, at least for various season, namely: the boundaries of forest, lands, banks of rivers and water bodies basing on the form [37] and color-texture domains [38,39]. An example is given in (see Figure 3) below.
- It should be noted that the use of the above-mentioned approaches for navigation requires, on the one hand, the solution of the camera calibration problems and the elimination of all kinds of registration nonlinearities, such as distortion [40,41,42], motion blurring [43,44], but the most important peculiarity is the registration of images on 2D photodetector array, that is, the transformation of the 3D coordinates of the object into 2D, which gives only the angular coordinates of objects, known in the literature as bearing-only observations [45]. This is special area of nonlinear filtering problem, which may be solved more or less successfully with the aid of linearized or extended Kalman filtering and also with particle and unscented Kalman filtering. Meanwhile the comparison of various filtering solution shows [46,47] either the presence of uncontrolled bias [48] or the urgent necessity of the filter dimension extension like for particle and unscented filtering. Meanwhile comparison of the filtering accuracy shows almost identical accuracy [49], that is why one should prefer most simple pseudomeasurement Kalman filter without bias, developed on the basis of Pugachev’s conditionally optimal filtering [50,51,52,53]. To obtain 3D coordinates, it is also possible to measure the range, which is possible using stereo systems [54,55,56] or using active radio or laser range finders [6]. The latter can be limited in use because they need essential power and disclose the UAV position, and the stereo systems require very accurate calibration and need the creation of a significant triangulation base which is rather difficult to maintain on small-sized UAV in flight.
- The problem of observing bearings only has long been in the focus of the interests of nonlinear filtering specialists, since it leads to the problem of estimating the position from nonlinear measurements. In the paper [16], we described a new filtering approach using the pseudo-measurement method, which allows expanding the observation system, up to unbiased estimations of the UAV’s own position, on the basis of the determination of bearings of terrestrial objects with known coordinates. However, the filtering is not the only problem which arises in bearing-only observations. Another issue is the association of observed objects with their images on template. Here the various approaches based on RANSAC solutions are necessary [57], such as [58,59], but the most important is the fusion of the current position estimation with the procedure of outliers rejection [60], for details see [16].
- In addition, bearing monitoring requires knowledge of the position of the line of sight of the surveillance system, which is not determined by the orientation angles of the apparatus coming from the INS. That is why it is of interest to estimate the line of sight position from the evolution of the optical flow (OF) or projective matrices describing the transformation of images of terrain sections over two consecutive frames. In the case of visual navigation one needs also the set of angles, determining the orientation of the camera optical axis. The general model, developed for OF observation and describing the geometry of the observation is given in [61], and the corresponding filtering equations for the UAV attitude parameters have been obtained in [62]. These equations and models were tested with the aid of special software package [63] and the possibility of the estimate of coordinates and angular velocities of the UAV were successfully demonstrated in [64,65,66]. However, neither OF nor evolution of projective matrices give the exact values of angles determining the position of the line of sight but define rather the angular velocities, so the problem of the angles estimation remains and must be solved with aid of filtering.

### 2.3. The UAV Position and the Coordinates Velocities Estimation

The methods described above for measuring various parameters, associated with the UAV movement supply different information, which must be appropriately converted into the inputs of the control system. In particular, the data on the coordinate velocities of the UAV motion are contained in the OF measurements and are extracted by filtering the dynamics equations with the corresponding measurements [65,67]. More difficult is the use of bearing-only measurements, although a complete set of filtering equations is given in the works [11,16,53]. In addition, observations of moving target with aid of bearing-only observations allow us to evaluate their velocities, which is shown in the work [68].

Filtering equations for the UAV velocities on the basis of the OF measurements have been given in [64,69] with examples of the estimation of the current altitude and coordinate velocity of straightforward motion.

### 2.4. The UAV Angles and Angular Velocities Estimation

In general, the OF field contains the information about the coordinate velocities of the UAV and angular velocities of the sight line. Reliable results were obtained on virtual series of images modelling the flight with constant coordinate velocity and rotation along yaw angle [64]. However, experiments with the real video shows the high correlation level between different motions, for example between pitch angle and velocity of descent. Unfortunately, the measurement of the position of the line of sight in the UAV coordinate system, which is very precise in principle, is distorted by the own movement of the apparatus, since the UAV slopes are necessary for maneuvering and their separation from the angles of the line of sight is a very delicate problem. For example in experiments with quadrocopter equipped with stabilized camera, one needed to distinguish the angle of the camera inclination and the vehicle inclination which is necessary for UAV motion itself. In our experiments without careful determination of the UAV inclination angle we did not get any reliable results related to the coordinate and vertical UAV motion [61,62,63].

#### Estimation of the Angular Position

UAV angular position estimation is given by three angles $\theta \left(t_{k}\right),\varphi \left(t_{k}\right),\gamma \left(t_{k}\right)$ (pitch, roll and yaw, respectively), angular velocities $\omega_{p}\left(t_{k}\right),\omega_{r}\left(t_{k}\right),\omega_{y}\left(t_{k}\right)$ and angular accelerations $a_{p}\left(t_{k}\right),a_{r}\left(t_{k}\right),a_{y}\left(t_{k}\right).$

Pitch angle and pitch angular velocity dynamics described by the following relations:

$$\begin{array}{c} \theta \left(t_{k+1}\right)=\theta \left(t_{k}\right)+\omega_{p}\left(t_{k}\right)\Delta t+a_{p}\left(t_{k}\right)\frac{\Delta t^{2}}{2},\\ \omega_{p}\left(t_{k+1}\right)=\omega_{p}\left(t_{k}\right)+a_{p}\left(t_{k}\right)\Delta t+W_{p}\left(t_{k}\right). \end{array}$$

where $W_{p}\left(t_{k}\right)$— is the white noise with variance $\sigma_{p}^{2}.$

The pitch angular velocity measurement using the OF has the following form:

$$\begin{array}{c} m_{p}\left(t_{k}\right)=\omega_{p}\left(t_{k}\right)+W_{\omega_{p}}\left(t_{k}\right), \end{array}$$

where $W_{\omega_{p}}\left(t_{k}\right)$— is the noise in the angular velocity measurements using OF, which is the white noise with variance $\sigma_{\omega_{p}}^{2}.$

Similarly to the coordinate velocity estimation we get the pitch angle $\theta (t_{k})$ and pitch angular velocity $\omega_{p}\left(t_{k}\right)$ estimations:

(2) $$\begin{array}{c} \hat{\theta }\left(t_{k+1}\right)=\hat{\theta }\left(t_{k}\right)+{\hat{\omega }}_{p}\left(t_{k}\right)\Delta t+a_{p}\left(t_{k}\right)\frac{\Delta t^{2}}{2}\\ {\hat{\omega }}_{p}\left(t_{k+1}\right)=K_{p}\left(t_{k+1}\right)m_{p}\left(t_{k+1}\right)+\left(1-K_{p}\left(t_{k+1}\right)\right)\left({\hat{\omega }}_{p}\left(t_{k}\right)+a_{p}\left(t_{k}\right)\Delta t\right),\\ K_{p}\left(t_{k+1}\right)=\frac{{\hat{P}}^{\omega_{p}\omega_{p}}\left(t_{k}\right)+\sigma_{p}^{2}}{{\hat{P}}^{\omega_{p}\omega_{p}}\left(t_{k}\right)+\sigma_{p}^{2}+\sigma_{\omega_{p}}^{2}},\\ {\hat{P}}^{\omega_{p}\omega_{p}}\left(t_{k+1}\right)=\frac{\sigma_{\omega_{p}}^{2}\left({\hat{P}}^{\omega_{p}\omega_{p}}\left(t_{k}\right)+\sigma_{p}^{2}\right)}{{\hat{P}}^{\omega_{p}\omega_{p}}\left(t_{k}\right)+\sigma_{p}^{2}+\sigma_{\omega_{p}}^{2}}. \end{array}$$

The formulae for $\hat{\varphi },\hat{\gamma }$ and $\hat{\omega_{r}},\hat{\omega_{y}}$ are analogous and used in the model based on the OF estimation.

## 3. Joint Estimation of the UAV Attitude with the Aid of Filtering

### 3.1. Visual-Based Navigation Approaches

Several studies have demonstrated the effectiveness of approaches based on motion field estimation and feature tracking for visual odometry [70]. Vision based methods have been proposed even in the context of autonomous landing management [12]. In [47] a visual odometry based on geometric homography was proposed. However, the homography analysis uses only 2D reference points coordinates, though for evaluation of the current UAV altitude the 3D coordinates are necessary. All such approaches presume the presence of some recognition system in order to detect the objects nominated in advance. Examples of such objects can be special buildings, crossroads, tops of mountains and so on. The principal difficulties are the different scale and aspect angles of observed and stored images which leads to the necessity of huge templates library in the UAV control system memory. Here one can avoid this difficulty, by using another approach based on observation of so-called feature points [71] that are the scale and the aspect angle invariant. For this purpose the technology of feature points [23] is used. In [10] the approach based on the coordinates correspondence of the reference points observed by on-board camera and the reference points on the map loaded into UAV’s memory before the mission start had been suggested. During the flight these maps are compared with the frame of the land, directly observed with the help of on-board video camera. As a result one can detect current location and orientation without time-error accumulation. These methods are invariant to some transformations and also are noise-stable so that predetermined maps can be different in scale, aspect angle, season, luminosity, weather conditions, etc. This technology appeared in [72]. The contribution of work [16] is the usage of modified unbiased pseudomeasurements filter for bearing only observations of some reference points with known terrain coordinates.

### 3.2. Kalman Filter

In order to obtain metric data from visual observations one needs first to make observations from different positions (i.e., triangulation) and then to use nonlinear filtering. However, all nonlinear filters either have unknown bias [48] or are very difficult for on-board implementation like the Bayesian type estimation [27,73]. Approaches for a position estimation based on bearing-only observations had been analyzed long ago especially for submarine applications [49] and nowadays for UAV applications [46].

Comparison of different nonlinear filters for bearing-only observations in the issue of the ground-based object localization [74] shows that EKF (extended Kalman filter), unscented Kalman filter, particle filter and pseudomeasurement filter give almost the same level of accuracy, while the pseudomeasurement filter is usually more stable and simple for on-board implementation. This observation is in accordance with older results [49], where all these filters were compared in the issue of moving objects localization. It has been mentioned that all these filters have bias which makes their use in data fusion issues rather problematic [45]. The principle requirement for such filters in data fusion is the non-biased estimate with known mean square characterization of the error. Among the variety of possible filters the pseudomeasurement filter can be easily modified to satisfy the data fusion demands. The idea of such nonlinear filtering has been developed by V. S. Pugachev and I. Sinitsyn in the form of so-called conditionally-optimal filtering [50], which provides the non-biased estimation within the class of linear filters with the minimum mean squared error. In our previous works we developed such a filter (so called Pseudomeasurement Kalman Filter (PKF)) for the UAV position estimation and give the algorithm for path planning along with the reference trajectory under external perturbations and noisy measurements [16,53].

### 3.3. Optical Absolute Positioning

Some known aerospace maps of a terrain in a flight zone are loaded into the aircraft memory before a start of flight. During the flight these maps are compared with the frame of the land, directly observed with the help of on-board video camera. For this purpose the technology of feature points [23] is used. As a result one can detect current location and orientation without time-error accumulation. These methods are invariant to some transformations and also are noise-stable so that predetermined maps can vary in height, season, luminosity, weather conditions, etc. Also from the moment of previous plane surveying the picture of this landscape can be changed due to human and natural activity. All approaches based on the capturing of the objects assigned in advance presume the presence of some on-board recognition system in order to detect and recognise such objects. Here we avoid this difficulty by using the observation of feature points [71] that are the scale and the aspect angle invariant. In addition, the modified pseudomeasurements Kalman Filtering (PKF) is used for estimation of UAV positions and control algorithm.

**Figure 4:**
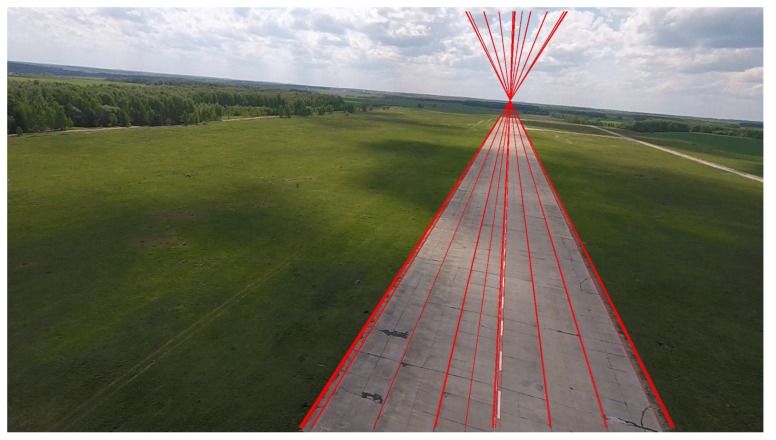
Landing on runway with well-structured epipolar lines.

One should mention also the epipolar position estimation for absolute positioning [75], where it helps at landing on runway (see Figure 4).

## 4. Projection Matrices Techniques for Videonavigation

The transformation of images of plane regions when the camera position is changed is described by a projective transformation given by the corresponding matrix. The complete matrix of the projective transformation contains information on the displacement of the main point of the lens and the rotation of the line of sight. Once installed on an aircraft flying over a relatively flat portion of the earth’s surface, the camera registers a sequence of frames, and if there is overlap between consecutive frames, analysis of the displacement of characteristic points in the overlap region carries information about the linear and angular motion of the camera and thereby about the UAV motion. Projective transformations are often used to match images, but here we use alignment for motion analysis. Although it is quite natural, here is the first time when we used it to estimate UAV motion using actual video survey data. The OF gives the estimation via measurement of coordinate velocities of the image shift and therefore provides just local esimations where angular components are highly correlated with coordinate velocities and the latter are few orders higher than angular ones. So the issue of estimation of angular velocities on the basis of OF looks very difficult. At the same time the estimation on the basis of projective matrix evolution looks more promising for estimation of angles of the sight line [76]. The estimation of motion via projective matrices have been known long ago [77] and remains in the focus of researchers until now [9,78,79]. The OF works only on the basis of local information on the speed of motion, which leads to the error drift incresing. Therefore, it would be useful to obtain corrective data about the orientation of the UAV at some intermediate time instances. Such information maybe obtained with the aid of projective transformation between successive frames.

Of course this method is known for a long time, however, until now in the literature we did find only few examples of using this method for the UAV navigation, see for example [80]. Perhaps the reason is that this method needs a good estimation of the initial position and the line of sight angles, therefore the fusion with filtering algorithms taking into account the dynamical model of the UAV is rather urgent. The artcile [80] presents also the estimation of errors, though they depend on the specific landscape features, so it would be nice to estimate the influence of the projective matrices computation on the accuracy of the shift and rotation evaluation. Here we also present the experimental results of the UAV position estimation based on the computation of projective matrices on the basis of real video data in combination with the filtering of coordinates and angles. The basic idea of our approach is similar to [81], though in our implementation of homography between two successive frames we add the estimation of motion via Kalman filtering. In [80] an interesting application of the UAV observation for road traffic where it is demonstrated the application of the projective transform to estimation of the vehicle velocity by analysis of two successive frames. The disadvantage of this approach is that it is based on knowledge of coordinates of some specific points within field of view. Generally such points are absent. Very interesting example of projective matrix technique usage is given in [82]. However, all these approaches look like successful applications in rather specific cases. General approach to the camera pose estimation has been presented in [81] and our algorithm follows to it basically. The principal novelty is the fusion of projection technique with Kalman filtering for angular position of sight line. It is the more so important since other techniques do not estimate the angular position of the camera directly and the angular position of camera appears implicitly only. The information about the direction of the line of sight is important in the usage of the OF as a sensor of linear and angular velocities especially if the UAV performs manoeuvres with large changes of roll angle and pitch angle. The projection matrix techniques look rather promising in fusion with the OF, however this approach needs further experiments and additional flights.

### 4.1. Coordinate Systems

#### 4.1.1. Earth Coordinate System

We assume the flat earth surface, the coordinate system OXYZ is chosen as follows:

- The origin *O* belongs to the earth surface.
- Axis OX is directed to the east.
- Axis OY is directed to the north.
- Axis OZ is directed to the zenith.

The earth surface is described by equation z=0.

#### 4.1.2. The Camera Coordinate System

The pinhole camera model is used and the following camera coordinate system $O^{\prime }X^{\prime }Y^{\prime }Z^{\prime }$ where

- $O^{\prime }$ is the principal point;
- axis $O^{{}^{\prime }}X^{{}^{\prime }}$ is directed right of the image;
- axis $O^{{}^{\prime }}Y^{{}^{\prime }}$ is directed to the top of the image;
- and axis $O^{{}^{\prime }}Z^{{}^{\prime }}$ is directed strightforwardly along the optical axis of the camera,

then coordinates of point $r^{\prime }=\left|\begin{array}{c} x^{\prime }\\ y^{\prime }\\ z^{\prime } \end{array}\right|$ in the camera coordinate system related with homogeneous coordinates in pixels $p=\left|\begin{array}{c} p_{x}\\ p_{y}\\ p_{z} \end{array}\right|$ of its image via the camera matrix *C* by relation

$$p=Cr^{\prime }.$$

**Figure 5:**
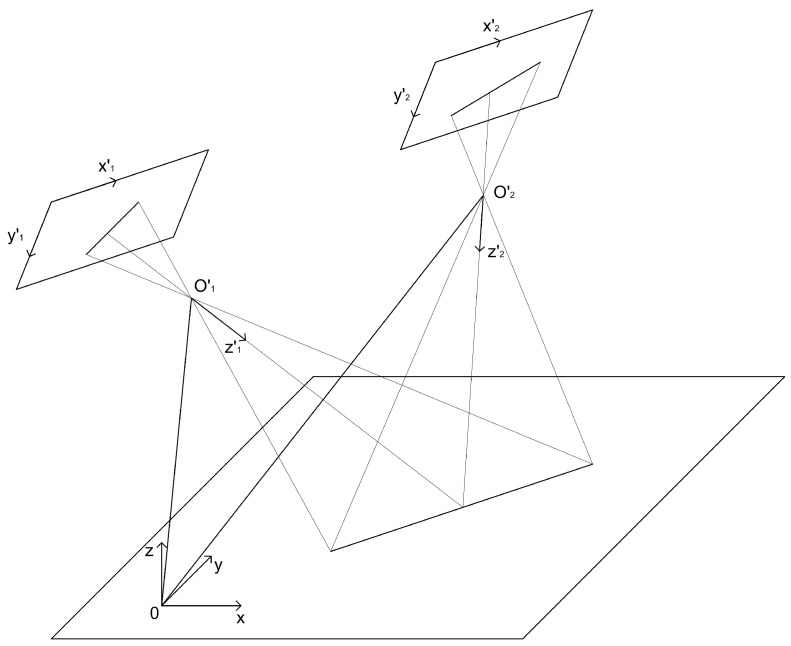
Coordinate system of two successive frames positions used for determining the projective matrix giving the relation between coordinates of singular points.

The coordinate systems are shown on Figure 5.

#### 4.1.3. Position of the Camera

Let at some moment the principal point of camera $O^{\prime }$ is represented in the earth coordinate system OXYZ by vector t. Then the coordinates of some point in the camera coordinate system $r^{\prime }$ and in the earth coordinate system $r=\left|\begin{array}{c} x\\ y\\ z \end{array}\right|$ related by formula

$$r^{\prime }=R\left(r-t\right),$$

where *R* is the matrix of the camera rotation, such that $R^{T}R=RR^{T}=E$.

So we have

p=CR(r−t).

#### 4.1.4. Representation of the Camera Rotation with the Aid of Roll, Pitch, Yaw Angles

For practical reasons the position of the camera can be defined by superposition of three rotations corresponding to the rotations of the vehicle, such as:

- Yaw that is rotation about the axis $O^{\prime }Z^{\prime }$ so that positive angle corresponds the anticlockwise rotation.
- Pitch that is rotation about the axis $O^{\prime }X^{\prime }$ so that for positive angle image moves downward.
- Roll that is rotation about the axis $O^{\prime }Y^{\prime }$ so that positive angle image moves right.

If the optical axis of the camera directed downward all rotations are zeros and the top of image is directed to the north.

### 4.2. Projective Representation of the Frame-to-Frame Transformation

Let us find the matrix of the projective transformation *P* between homogeneous coordinates on the earth surface $\rho =\left|\begin{array}{c} x\\ y\\ 1 \end{array}\right|$ and coordinates p of the frame pixels

p=Pρ.

Denote as $M^{\left[1,2\right]}$ the matrix comprising first two columns of M, that is

$$M^{\left[1,2\right]}=M\left|\begin{array}{cc} 1 & 0\\ 0 & 1\\ 0 & 0 \end{array}\right|.$$

$$p=CR\left(r-t\right)=CR\left(\left|\begin{array}{c} x\\ y\\ 0 \end{array}\right|-t\right)=C\left(R\left|\begin{array}{c} x\\ y\\ 0 \end{array}\right|-Rt\right)$$

$$=C\left(R^{\left[1,2\right]}\left|\begin{array}{c} x\\ y \end{array}\right|-Rt\right)=C\left[\;R^{\left[1,2\right]},\;-Rt\;\right]\rho .$$

Therefore,

$$P=C\left[\;R^{\left[1,2\right]},\;-Rt\;\right].$$

### 4.3. Determining of the Camera Position

Assume we have two positions of the camera, where

- the first one is known $R_{1}$, $t_{1}$.
- the second one to be determined $R_{2}$, $t_{2}$.

Then

- $p_{1}=CR_{1}\left(r-t_{1}\right),\;\;\;P_{1}=C\left[\;R_{1}^{\left[1,2\right]},\;-R_{1}t_{1}\;\right],\;\;\;p_{1}=P_{1}\rho$.
- $p_{2}=CR_{2}\left(r-t_{2}\right),\;\;\;P_{2}=C\left[\;R_{2}^{\left[1,2\right]},\;-R_{2}t_{2}\;\right],\;\;\;p_{2}=P_{2}\rho$.

And therefore,

$$p_{2}=P_{2}P_{1}^{-1}p_{1}.$$

From other side, $p_{2}=Hp_{1}$, where *H* is the matrix of projective transformation of frame 1 to frame 2, so we get

$$H=P_{2}P_{1}^{-1}=\left(C\left[\;R_{2}^{\left[1,2\right]},\;-R_{2}t_{2}\;\right]\right){\left(C\left[\;R_{1}^{\left[1,2\right]},\;-R_{1}t_{1}\;\right]\right)}^{-1}$$

$$=C\left[\;R_{2}^{\left[1,2\right]},\;-R_{2}t_{2}\;\right]{\left[\;R_{1}^{\left[1,2\right]},\;-R_{1}t_{1}\;\right]}^{-1}C^{-1},$$

$$C^{-1}HC=\left[\;R_{2}^{\left[1,2\right]},\;-R_{2}t_{2}\;\right]{\left[\;R_{1}^{\left[1,2\right]},\;-R_{1}t_{1}\;\right]}^{-1},$$

$$C^{-1}HC\left[\;R_{1}^{\left[1,2\right]},\;-R_{1}t_{1}\;\right]=\left[\;R_{2}^{\left[1,2\right]},\;-R_{2}t_{2}\;\right],$$

**Figure 6:**
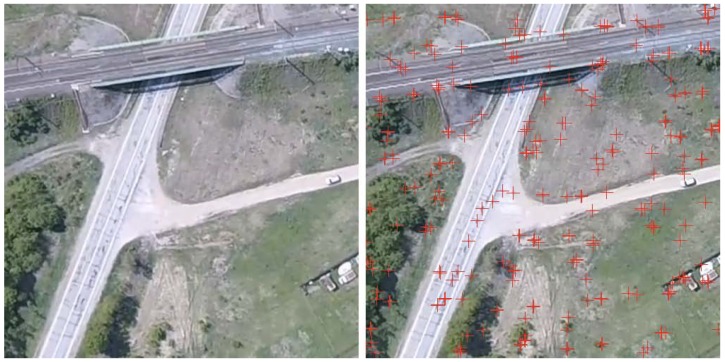
The two successive frames used for determining the projective matrix. Red crosses show the singular points usedw for the matrix calculation. The difference between two frames is rather small since it corresponds to time interval Δt=1/25 s. The low resolution is due to the aircraft motion, which produce additional blurring.

Any matrix obtained from *H* by multiplying on k≠0, that is kH, determines the same projective transformation. Assume we got the estimate $\hat{H}$ of the matrix kH obtained on the basis of two successive frames. An example of the matrix *H* obtained on the basis of two successive frames like in Figure 6 is shown below.

Example of calculated matrix *H* between two successive frames

$$H=\left(\begin{array}{ccc} 1.023 & 0.008753 & -4.014\\ -0.001305 & 1.010 & 70.08\\ 1.131\times 10^{-5} & 6.478\times 10^{-6} & 1.0 \end{array}\right)$$

For getting $\hat{H}$ we use the RANSAC methodology [57] and interpret the difference $\hat{H}-kH$ as a normal noise, so as $\hat{H}=kH+\varepsilon ,$ where ε is a matrix normal noise added to all entries of $\hat{H}.$ It permits to determine the second camera position as a solution of the following minimization problem:

(3) $$\left[{\hat{R}}_{2},{\hat{t}}_{2},\hat{k}\right]=\arg\min_{\left[R,t,k\right]}{\left|\left|C^{-1}\frac{\hat{H}}{k}C\left[\;R_{1}^{\left[1,2\right]},\;-R_{1}t_{1}\;\right]-\left[\;R^{\left[1,2\right]},\;-Rt\;\right]\right|\right|}_{F}^{2}.$$

or equivalently

(4) $$\left[{\hat{R}}_{2},{\hat{t}}_{2},\hat{k}\right]=\arg\min_{\left[R,t,k\right]}{\left|\left|C^{-1}\hat{H}C\left[\;R_{1}^{\left[1,2\right]},\;-R_{1}t_{1}\;\right]-k\left[\;R^{\left[1,2\right]},\;-Rt\;\right]\right|\right|}_{F}^{2}.$$

### 4.4. Solution of Minimization Problem

The minimization problem (3) or/and (4) admit the following solution. Introduce the matrix $G=C^{-1}\hat{H}C\left[\;R_{1}^{\left[1,2\right]},\;-R_{1}t_{1}\;\right]$. Then the above problem may be reformulated as follows

$$\left[{\hat{R}}_{2},{\hat{t}}_{2},\hat{k}\right]=\arg\min_{\left[R,t,k\right]}{\left|\left|G-k\left[\;R^{\left[1,2\right]},\;-Rt\;\right]\right|\right|}_{F}^{2}.$$

Denote as $M^{\left[3\right]}$ the third column of matrix *M* ($M^{\left[3\right]}=M\left|\begin{array}{c} 0\\ 0\\ 1 \end{array}\right|$) and by analogy denote $M^{\left[1\right]}$ and $M^{\left[2\right]}$. So the minimization problem may be rewritten as

$$\left[{\hat{R}}_{2},{\hat{t}}_{2},\hat{k}\right]=\arg\min_{\left[R,t,k\right]}\left({\left|\left|G^{\left[1,2\right]}-kR^{\left[1,2\right]}\right|\right|}_{F}^{2}+{\left|\left|G^{\left[3\right]}+kRt\right|\right|}_{2}^{2}\right).$$

The first term does not depend on *t*, the second term achieves its minimum at $t=-\frac{1}{k}R^{T}G^{\left[3\right]}$ where it is equal zero. Thus ${\hat{t}}_{2}=-\frac{1}{\hat{k}}{\hat{R}}_{2}^{T}G^{\left[3\right]}$. By substitution to the original minimizing term one can reduce the problem to the following

(5) $$\left[{\hat{R}}_{2},\hat{k}\right]=\arg\min_{\left[R,k\right]}{\left|\left|G^{\left[1,2\right]}-kR^{\left[1,2\right]}\right|\right|}_{F}^{2}.$$

Vectors $G^{\left[1\right]}$ and $G^{\left[2\right]}$ belong to some plane γ, therefore vectors ${\hat{R}}_{2}^{\left[1\right]}$ and ${\hat{R}}_{2}^{\left[2\right]}$ giving minimum in (5) belong to the same plane.

Introduce the orthonormal basis $V=\left[V^{\left[1\right]},V^{\left[2\right]},V^{\left[3\right]}\right]$, such that $V^{\left[1\right]},V^{\left[2\right]}\in \gamma$ define the system of coordinates on γ

$$V^{\left[1\right]}=\frac{G^{\left[1\right]}}{\left|\right|G^{\left[1\right]}{\left|\right|}_{2}},\;\;\;V^{\left[2\right]}=\frac{G^{\left[2\right]}-\left({V^{\left[1\right]}}^{T}G^{\left[2\right]}\right)V^{\left[1\right]}}{\left|\right|G^{\left[2\right]}-\left({V^{\left[1\right]}}^{T}G^{\left[2\right]}\right)V^{\left[1\right]}{\left|\right|}_{2}},\;\;\;V^{\left[3\right]}=V^{\left[1\right]}\times V^{\left[2\right]}.$$

In this system of coordinates γ vectors $G^{\left[1\right]}$ and $G^{\left[2\right]}$ have coordinates $\left[g^{\left[1\right]},g^{\left[2\right]}\right]=V^{-1}G^{\left[1,2\right]}$.

At the same time vectors ${\hat{R}}_{2}^{\left[1\right]}$ and ${\hat{R}}_{2}^{\left[2\right]}$ in the same system of coordinates have representation

$$\left[r^{\left[1\right]},r^{\left[2\right]}\right]=\left|\begin{array}{cc} \cos(\alpha ) & -\sin(\alpha )\\ \sin(\alpha ) & \cos(\alpha )\\ 0 & 0 \end{array}\right|.$$

So the problem is reduced to the determining of an angle $\hat{\alpha }$ and $\hat{k}$:

$$\left[\hat{\alpha },\hat{k}\right]=\arg\min_{\alpha ,k}{\left|\left|\left[g^{\left[1\right]},g^{\left[2\right]}\right]-k\left|\begin{array}{cc} \cos(\alpha ) & -\sin(\alpha )\\ \sin(\alpha ) & \cos(\alpha )\\ 0 & 0 \end{array}\right|\right|\right|}_{F}.$$

By differentiation with respect to α one can obtain that

$$\tan\left(\hat{\alpha }\right)=\frac{g_{2}^{\left[1\right]}-g_{1}^{\left[2\right]}}{g_{2}^{\left[2\right]}+g_{1}^{\left[1\right]}}$$

does not depend on k.

From this equation one can obtain two solutions, the second one will be rejected later.

Now one can obtain $\left[r^{\left[1\right]},r^{\left[2\right]}\right]$.

Later the rotation matrix be determined by transformation of its columns back to the original coordinates system

$${\hat{R}}_{2}=V\left[r^{\left[1\right]},\;\;\;r^{\left[2\right]},\;\;\;r^{\left[1\right]}\times r^{\left[2\right]}\right].$$

Then define $\hat{k}$ in accordance with linear regression with quadratic penalization

$$\hat{k}=\frac{{g^{\left[1\right]}}^{T}r^{\left[1\right]}+{g^{\left[2\right]}}^{T}r^{\left[2\right]}}{{r^{\left[1\right]}}^{T}r^{\left[1\right]}+{r^{\left[2\right]}}^{T}r^{\left[2\right]}},$$

finally, ${\hat{t}}_{2}=-\frac{1}{\hat{k}}{\hat{R}}_{2}^{T}G^{\left[3\right]}$. It appears that two solutions for ${\hat{t}}_{2}$ differs by the sign of *Z* coordinate, so the extra solution lies under earth and must be rejected.

### 4.5. Testing of the Algorithm

Suppose that we have a map, represented in raster(-scan) graphics, where $q=\left|\begin{array}{c} q_{x}\\ q_{y}\\ 1 \end{array}\right|$ are their pixel coordinates. They are related with the earth coordinates by matrix Q, that is

q=Qρ.

Let the length unit on the earth corresponds to *k* pixels on the map and the map size is *w* (width) and *h* (hight) pixels. For example the origin of the earth coordinate system *O* is in the map center. Then:

$$Q=\left|\begin{array}{ccc} k & 0 & w/2\\ 0 & -k & h/2\\ 0 & 0 & 1 \end{array}\right|.$$

Relation between the frame pixels $p_{i}$ and the map pixels *q* is given by

$$p_{i}=P_{i}\rho ,\;\;\;\rho =Q^{-1}q\;\;\;\Rightarrow \;\;\;p_{i}=P_{i}Q^{-1}q,$$

or

$$p_{i}=A_{i}q,\;\;\;A_{i}=P_{i}Q^{-1}=C\left[\;R_{i}^{\left[1,2\right]},\;-R_{i}t_{i}\;\right]Q^{-1}.$$

Note that

$$H=P_{2}P_{1}^{-1}=P_{2}\left(Q^{-1}Q\right)P_{1}^{-1}=\left(P_{2}Q^{-1}\right)\left(QP_{1}^{-1}\right)=\left(P_{2}Q^{-1}\right){\left(P_{1}Q^{-1}\right)}^{-1}=A_{2}A_{1}^{-1}.$$

Then the test algorithm is as follows

- Choose the map and find the connection with the earth coordinates by determining *Q*.
- Choose *C*.
- Choose $R_{i}$, $t_{i}$.
- Calculate $A_{i}=C\left[\;R_{i}^{\left[1,2\right]},\;-R_{i}t_{i}\;\right]Q^{-1}$.
- Forget for a moment $R_{2}$, $t_{2}$.
- Obtain two frames in accordance with $A_{i}$ from the map.
- Visually test that they correspond to $R_{i}$, $t_{i}$.
- Calculate the matrix $H=A_{2}A_{1}^{-1}$.
- Model $\hat{H}=kH+\varepsilon ,\;\;\;k\neq 0,\;\;\;\varepsilon$ is a noise.
- Find ${\hat{R}}_{2},{\hat{t}}_{2}$.
- Recall $R_{2}$, $t_{2}$.
- Compare ${\hat{R}}_{2}$ with $R_{2}$, and ${\hat{t}}_{2}$ with $t_{2}$.

#### 4.5.1. Testing Results

In this testing we are evaluating the influence of noise in determining of projective matrix on the camera motion estimation. Testing shows that

- in the case of the noise absence ε=0 in the matrix $\hat{H}$ the method gives exact values of rotation and shift ${\hat{R}}_{2}=R_{2}$, ${\hat{t}}_{2}=t_{2}$,
- an increasing of the noise level evidently produce the increasing of errors in estimation, though the exact estimations needs further research with additional flight experiments.

**Figure 7:**
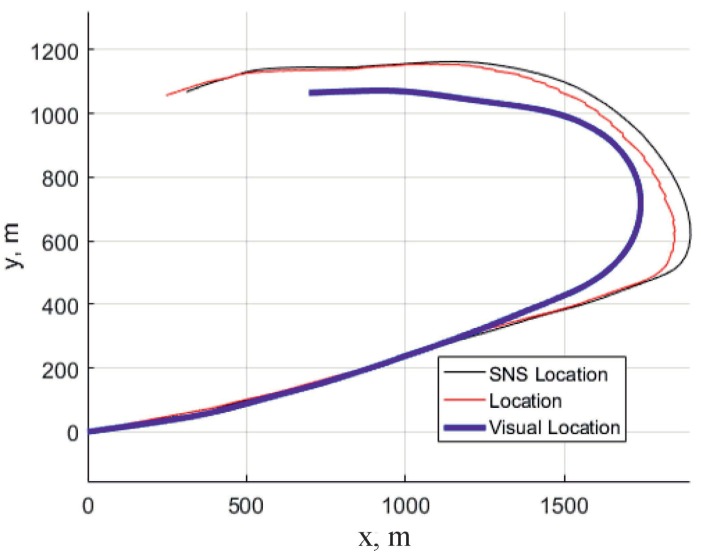
Solid line shows the position of the vehicle obtained from Satelite+ Navigation system (SNS). Blue line shows the position of the vehicle determined with the aid of the sequence of projective matrices. One can observe the increasing shift, since there is no correction based on the position evaluation.

**Figure 8:**
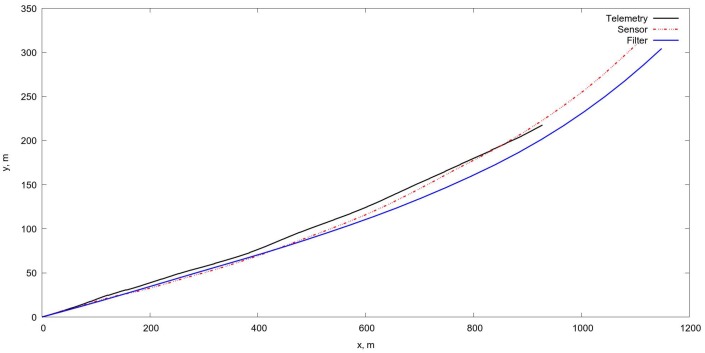
Tracking for X,Y coordinates. “Sensor” means estimation via projective matrices calculation.

**Figure 9:**
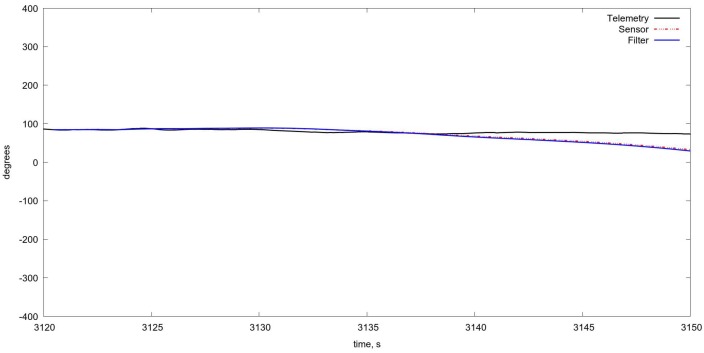
Tracking for yaw angle. The correspondence is rather good, but the flight does not contain yaw maneuvering.

**Figure 10:**
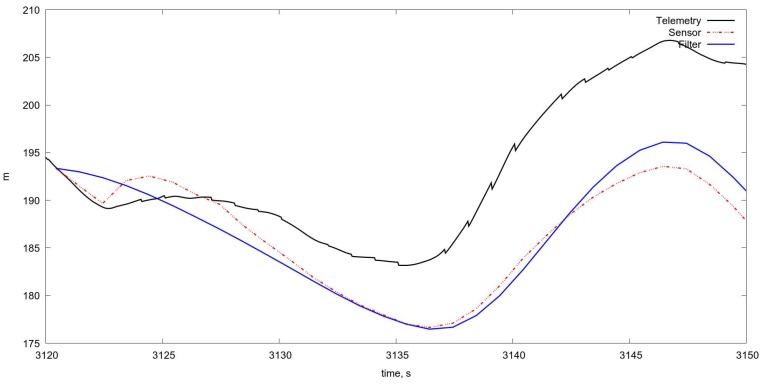
Tracking for *Z* coordinate. The correspondence is not bad on the very short interval at the beginning only, after that the error increases very fast.

An example of the algorithm testing on real flight data with the estimation of the UAV position on the basis of Kalman filtering is shown in Figure 7. More specific results showing the tracking of separate UAV motion components are shown also on Figure 8, Figure 9 and Figure 10.

They show more or less good correspondence but only on rather short time intervals, approximately 30 s; however, it is typical for algorithms giving the estimates of the UAV velocities, since all algorithms which involve accumulating of the drift need the correction with the aid of another algorithm. These algorithms should be based on matching of observed images and templates, some of them are described in Section 2 and Section 3.

#### 4.5.2. Statistical Analysis of Projective Matrices Algorithm

Thus, the average error equals 3.1837 m per frame, where the average frame shift equals 16.8158 m. The average relative error/per frame equals ≈19.08. See the Table 1.

#### 4.5.3. Comparison of Projective Matrices Algorithm with OF Estimation

Both projective matrices algorithms and the OF give infromation related to the coordinate and the angular velocities of the UAV. We test the OF approach [63] on the same videodata as for projective matrices.

**Figure 11:**
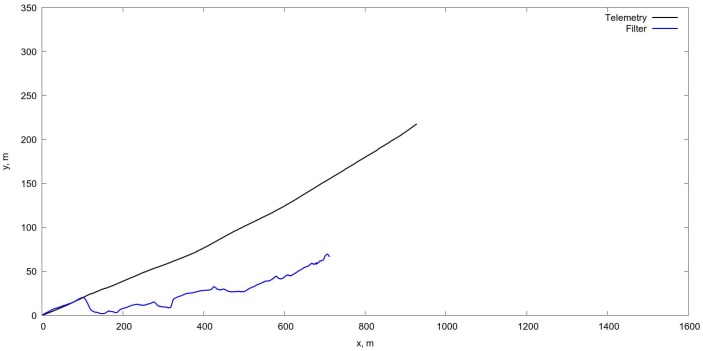
Tracking for X,Y coordinates via OF algorithm. In comparison with Figure 8 one can observe very short period of reliable tracking.

**Figure 12:**
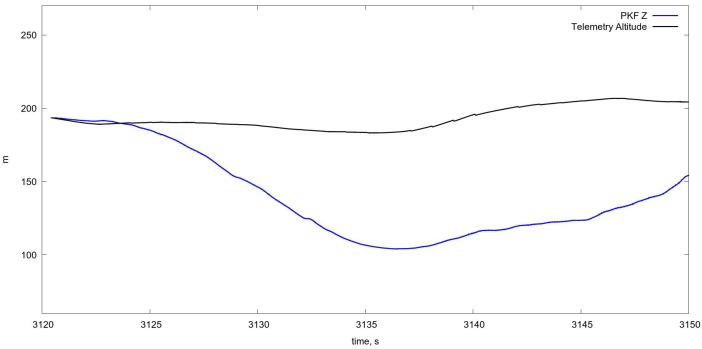
Tracking for *Z* coordinate via OF algorithm. In comparison with Figure 9 one can observe very short period of reliable tracking.

**Figure 13:**
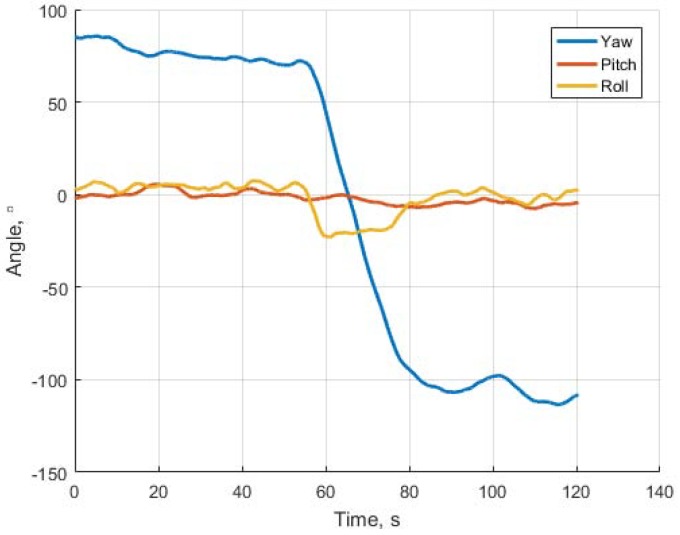
Orientation angles of the UAV estimated via projective matrices algorithm. One can observe the turn in yaw angle which the plane performed between 60-th and 80-th seconds of flight. On Figure 7 it corresponds approximately 1700 m from the starting point. At the initial period the estimate of yaw angle is around $85^{o}$ (see also Figure 9.)

On Figure 11 and Figure 12 in comparison with corresponding data on Figure 8 and Figure 9 one can see a very short period of reliable estimation of coordinates, so the projective matrices computation shows more relible tracking, however it is just a comparison of the algorithms per se, without fusion with INS, which is necessary in order to evaluate the current flight parameters. The estimation of the orientation angles is given in Figure 13.

The described algorithm gives only one step in the estimation of the displacement and rotation of the camera, while the initial data for the operation of the algorithm must be obtained from the system for estimating the position of the apparatus. In other words, the algorithm based on the calculation of the projective transformation from frame to frame can complement the sensors of the coordinate and angular velocities of the apparatus and its performance depends on the accuracy of the position matching of the singular points. Of course, this modelling example needs further verification with new videodata and new telemetry data from INS. It is obvious that the noise in the definition of the projective transformation matrix is decisive in assessing the operability of the algorithm and depends on a variety of factors. Therefore, further analysis of the algorithm will be performed on the new flight data sets. The matherial presented in this Section 4 provides a complex tracking algorithm in which the computation of the projective transformation serves as a sensor of displacement and rotations of the line of sight. Moreover, with the example of a sufficiently long flight, the possibility of determining the UAV velocities is shown on the basis of the algorithm for calculating a projective transformation from frame to frame. This demonstrates the possible efficiency of the approach and opens the way for its integration in the navigation system. Anyway the efficiency of this algorithm strongly depends on the other systems giving, for example, the initial estimates of position and angles, otherwise the increasing errors are inevitable. It is clear that the realization of video navigation from observations of the earth’s surface is a difficult task and we are only at the very beginning of the road.

## 5. Conclusions

The article describes a number of approaches to video navigation based on observation of the earth’s surface during an autonomous UAV flight. It should be noted that their performance depends on external conditions and the observed landscape. Therefore, it is diffcult to choose the most promising approach in advance and most likely it is necessary to rely on the use of various algorithms taking into account the enviromental conditions and computational and energy limitations of real UAV. However, there is an important problem, namely, the determination of the quality of the evaluation and the selection of the most reliable observation channels during the flight. The theory of control of observations opens the way to a solution, since the theory of filtering, as a rule, uses a discrepancy between the predicted and observed values and videos could help to detect them. This is probably, most of all, the main direction of future research in the field of video integration with standard navigation systems.

## Figures and Tables

**Table 1:** Analysis of the shift estimation provided by projective matrices algorithm.

Number of Frame Δt=0.5 s	$L_{2}$ Norm of Real Shift from INS	$L_{2}$ Norm of the Error from INS and Projective Matrices
1	17.9950	0.5238
2	16.6470	2.2434
3	17.4132	0.9915
4	16.4036	3.2009
5	17.2792	5.2980
6	16.2951	3.7480
7	17.7030	6.7479
8	16.4029	4.8240
9	17.8309	0.1152
10	16.1718	3.8627
11	17.2313	2.6757
12	15.8342	6.7456
13	17.2652	2.1774
14	15.7978	1.5271
15	17.4473	2.2599
16	15.9407	3.8367
17	17.4862	5.3902
18	16.0020	1.9714
19	17.2286	2.1162
20	15.9406	3.4193
